# Aberrant cortical–subcortical-cerebellar connectivity in resting-state fMRI as an imaging marker of schizophrenia and psychosis: a systematic review of data-driven whole-brain functional connectivity analyses

**DOI:** 10.3389/fnimg.2025.1650987

**Published:** 2025-10-10

**Authors:** Kyle M. Jensen, Tricia Z. King, Pablo Andrés-Camazón, Vince D. Calhoun, Armin Iraji

**Affiliations:** ^1^Department of Psychology, Georgia State University, Atlanta, GA, United States; ^2^Tri-Institutional Center for Translational Research in Neuroimaging and Data Science (TReNDS), Atlanta, GA, United States; ^3^Nell Hodgson Woodruff School of Nursing, Emory University, Atlanta, GA, United States; ^4^Institute of Psychiatry and Mental Health, Hospital General Universitario Gregorio Marañón, IiSGM, CIBERSAM, ISCIII, School of Medicine, Universidad Complutense, Madrid, Spain; ^5^Department of Computer Science, Georgia State University, Atlanta, GA, United States; ^6^Neuroscience Institute, Georgia State University, Atlanta, GA, United States

**Keywords:** schizophrenia, psychosis, resting-state fMRI, cortical–subcortical-cerebellar, cerebello-thalamo-cortical, whole-brain, functional connectivity, data-driven

## Abstract

**Introduction:**

Schizophrenia is extremely heterogenous, and the underlying brain mechanisms are not fully understood. Many attempts have been made to substantiate and delineate the relationship between schizophrenia and the brain through unbiased exploratory investigations of resting-state functional magnetic resonance imaging (rs-fMRI). The results of numerous data-driven rs-fMRI studies have converged in support of the disconnection hypothesis framework, reporting aberrant connectivity in cortical–subcortical-cerebellar circuitry. However, this model is vague and underspecified, encompassing a vast array of findings across studies. It is necessary to further refine this model to identify consistent patterns and establish stable imaging markers of schizophrenia and psychosis. The organizational structure of the NeuroMark atlas is especially well-equipped for describing functional units derived through independent component analysis (ICA) and uniting findings across studies utilizing data-driven whole-brain functional connectivity (FC) to characterize schizophrenia and psychosis.

**Methods:**

Toward this goal, a systematic literature review was conducted on primary empirical articles published in English in peer-reviewed journals between January 2019–February 2025 which utilized cortical–subcortical-cerebellar terminology to describe schizophrenia-control comparisons of whole-brain FC in human rs-fMRI. The electronic databases utilized included Google scholar, PubMed, and APA PsycInfo, and search terms included (“schizophrenia” OR “psychosis”) AND “resting-state fMRI” AND (“cortical–subcortical-cerebellar” OR “cerebello-thalamo-cortical”).

**Results:**

Ten studies were identified and NeuroMark nomenclature was utilized to describe findings within a common reference space. The most consistent patterns included cerebellar-thalamic hypoconnectivity, cerebellar-cortical (sensorimotor & insular-temporal) hyperconnectivity, subcortical (basal ganglia and thalamic)—cortical (sensorimotor, temporoparietal, insular-temporal, occipitotemporal, and occipital) hyperconnectivity, and cortical–cortical (insular-temporal and occipitotemporal) hypoconnectivity.

**Discussion:**

Patterns implicating prefrontal cortex are largely inconsistent across studies and may not be effective targets for establishing stable imaging markers based on static FC in rs-fMRI. Instead, adapting new analytical strategies, or focusing on nodes in the cerebellum, thalamus, and primary motor and sensory cortex may prove to be a more effective approach.

## Introduction

Schizophrenia is a severe psychiatric disorder and major cause of disability worldwide (Theodoridou and Rössler, 2010). While much progress has been made over the last few decades to establish the biological profile of schizophrenia and understand the underlying neural mechanisms (Dabiri et al., 2022; Meyer-Lindenberg, 2010), much work is still needed to establish stable imaging markers with clinical applications (Insel and Cuthbert, 2015; Jablensky, 2010; Morris et al., 2022). The clinical presentation of schizophrenia is highly heterogenous, with a great amount of variability across individuals (Wolfers et al., 2018), although psychosis (e.g., hallucinations, delusions, and disorganized behavior and speech) is generally considered the most characteristic feature, and is consequently the focus of much research.

### Aberrant cortical–subcortical-cerebellar connectivity

Many researchers have utilized neuroimaging methods such as resting state functional magnetic resonance imaging (rs-fMRI) as a promising method for the development of reliable markers of schizophrenia. rs-fMRI has proven to be useful for this purpose as it avoids bias linked to specific tasks and is non-invasive and less intensive than modalities involving experimental tasks, making it ideal for clinical populations (Fu et al., 2024). These approaches have experienced rapid growth in recent years, reaching a record high in 2019 with more than 100 peer-reviewed articles published utilizing rs-fMRI to examine schizophrenia populations (Fu et al., 2024). However, like individual symptom profiles, the reported patterns of aberrant functional connectivity (FC) derived from rs-fMRI are also heterogenous (Jiang et al., 2013). The disconnection hypothesis of schizophrenia (Friston, 1998) has been widely applied to help interpret these findings, postulating that schizophrenia reflects a dysfunctional integration of neuronal activity. The specific patterns of dysconnectivity have often been characterized by Andreasen et al. (1998) theory of cognitive dysmetria which poses that symptoms of schizophrenia arise from disruptions in cortical–subcortical-cerebellar circuitry. In the last 20–30 years this framework has become more established, supported by a wide body of research (Bernard and Mittal, 2015; Friston et al., 2016; Harikumar et al., 2023; Hwang et al., 2022; Li et al., 2019; Wei et al., 2025).

Although Andreasen et al. (1998) originally emphasized disruptions in circuitry between the cerebellum, thalamus, and prefrontal cortex, this framework has become somewhat of an underspecified umbrella term encompassing many different findings spanning the whole brain. For example, Matsuo et al. (2013) reports reduced activation in the inferior frontal gyrus, thalamus, and cerebellum, while Walther et al. (2017) reports hyperconnectivity between the motor cortex and thalamus, between the motor cortex and cerebellum, and between the subthalamic nucleus and anterior cingulate and dorsolateral prefrontal cortex. Yao et al. (2025) uses a slightly more specific variation of the term, “cerebello-*thalamo*-cortical,” to describe disruptions in circuitry between the cerebellum and postcentral gyrus, between the thalamus and middle temporal gyrus, and between the thalamus and middle and inferior occipital gyri. “Cerebello-*thalamo*-cortical” centers the model specifically on the thalamus and emphasizes its modulatory role (Harikumar et al., 2023; Hwang et al., 2022), however, this model still lacks specificity as the thalamus is a central hub in many brain networks (Hwang et al., 2017). Importantly, the findings of these three studies are largely non-overlapping, and yet they all use variations of “cortical–subcortical-cerebellar” to describe their findings. While this framework has proven to be useful in helping to overcome cortico-centric bias (Parvizi, 2009) by incorporating subcortical and cerebellar structures into pathological models of schizophrenia and psychosis, these heuristics overly generalize findings and should be supplemented with more descriptive terms, such as directionality and specific subcortical and cortical structures, if the field is to establish stable and reliable imaging markers. This lack of specificity may be reflective of a broader challenge facing the field of neuroscience, which is a lack of standardization for describing brain networks.

### Addressing inconsistency and heterogeneity with NeuroMark

Perhaps due to the highly interdisciplinary nature of the field, or because it is a relatively young branch of science, a disinclination to articulate findings through clear and consistent nomenclature has been identified as a major weakness within the field of neuroscience (Uddin et al., 2019, 2023). While there are many factors potentially contributing to these inconsistencies, individual subject variability across subjects within the same study (see Figure 1 in Jensen et al., 2024b) as well as within a single subject over the course of an fMRI scan (Iraji et al., 2019) only complicates the issue further. Thus, there is great benefit in efforts toward standardization, such as those employed by the NeuroMark approach (Du et al., 2020; Iraji et al., 2023; Jensen et al., 2024b), which employs spatially-constrained (Lin et al., 2010) independent component analysis (ICA; Calhoun et al., 2001) to incorporate spatial priors or templates derived from large datasets to identify functional units known as intrinsic connectivity networks (ICNs). These ICNs are sensitive to subject, dataset, and study level differences and have been adapted into a common reference space known as the NeuroMark 2.2 atlas (Jensen et al., 2024b). The NeuroMark 2.2 atlas has further improved the accessibility and interpretability of findings across studies by describing ICNs in terms familiar to the fields of cognitive and affective neuroscience (Jensen et al., 2024b). This template consists of 105 ICNs which cover the whole brain (see Table 1 and Figure 2 in Jensen et al., 2024b), incorporate information from multiple spatial scales, and have demonstrated reliability across the lifespan (Bajracharya et al., 2024).

Whole-brain data-driven functional connectivity methods, such as those utilizing group ICA (Calhoun et al., 2001), allow for unbiased exploratory approaches which yield rich and comprehensive results (Calhoun et al., 2009). However, the large amount of information produced by data-driven approaches such as these can be a double-edged sword. While they have great potential to facilitate valuable new discoveries, one of the challenges inherent with data-driven approaches lies in tasks of organizing, summarizing, and synthesizing vast amounts of information (Calhoun et al., 2021; Hutchison et al., 2013). Unfortunately, the challenge of interpreting these findings is a burden which is often placed on the reader (Allen et al., 2012). Furthermore, it can be difficult to compare findings across studies employing blind ICA due to variations in the identified ICNs (Abou-Elseoud et al., 2010; Du et al., 2020). Such inconsistencies across studies contribute to the heterogeneity currently hindering the development of a coherent biologically-informed model of schizophrenia.

The objective of the current review was to identify a collection of data-driven whole-brain studies within recent rs-fMRI literature which examine and describe group differences between individuals with schizophrenia or psychosis and controls within a cortical–subcortical-cerebellar framework and then translate their findings into a common reference space. The review focused on studies which utilized ICA in delineating their regions of interest (ROIs) and which were published within the last 5 years (2019-present) to capture studies published within approximately the same time frame since the NeuroMark approach was first developed and implemented, as comparability with the NeuroMark framework was an integral part of the review. By comparing findings across studies within the unifying framework of NeuroMark 2.2, the review aimed to inform the development of stable imaging markers of schizophrenia by determining which specific patterns of dysconnectivity were most consistent in studies referencing the cortical–subcortical-cerebellar and cerebello-thalamo-cortical framework. The review also sought to compare potentially relevant features which may help to explain inconsistencies across studies.

## Methods

This systematic review was conducted according to the Preferred Reporting Items for Systematic reviews and Meta-Analyses (PRISMA) (Page et al., 2021).

### Eligibility criteria

The review sought to include primary empirical articles published in English in peer-reviewed journals in the last 5 years reporting analyses of human resting-state fMRI and utilizing cortical–subcortical-cerebellar terminology to describe schizophrenia or psychosis. All studies were required to include schizophrenia subjects in their clinical sample as that was the primary interest of the current review, however, other clinical profiles were included in the final article selection if they were part of a larger psychosis sample because much of the schizophrenia literature is confounded with psychosis. In addition, to maximize the comparability of findings, this review only included studies with data-driven case–control comparisons of whole-brain functional connectivity. The review sought to further refine its inclusion of data-driven approaches by only including studies which implemented ICA in delineating their ROIs.

### Information sources, search strategy, and data extraction

**Figure 1:**
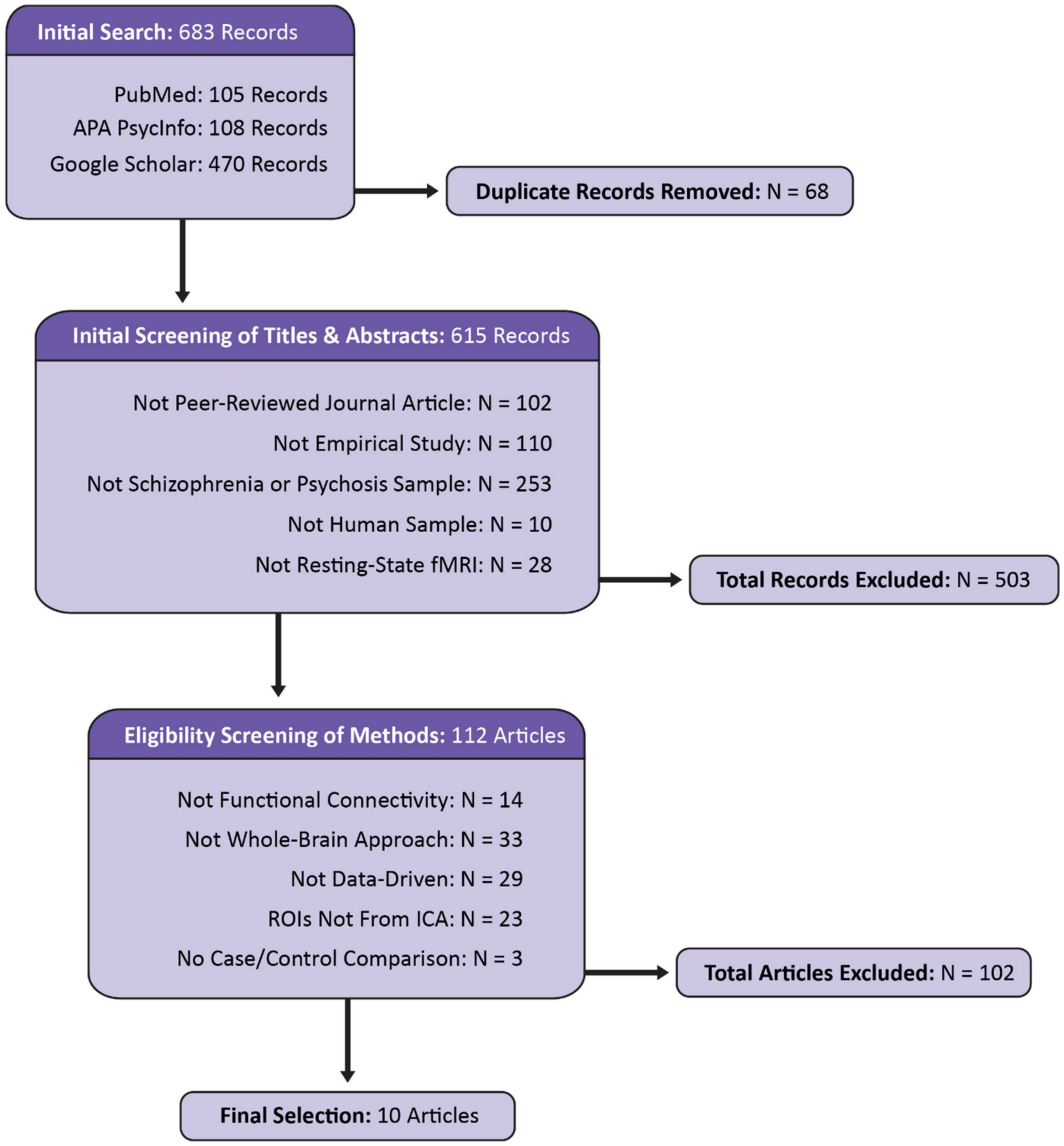
Flow diagram displaying the identification, screening, and selection of articles for the current review. Modeled after the PRISMA 2020 template (Page et al., 2021).

Included articles were identified using PubMed, APA PsycInfo, and Google Scholar. The search was restricted to a five-year period spanning January 1, 2019 to February 4, 2025. Search terms included: (“schizophrenia” OR “psychosis”) AND “resting-state fMRI” AND (“cortical–subcortical-cerebellar” OR “cerebello-thalamo-cortical”). The titles and abstracts of 683 identified records were screened for basic criteria (see Figure 1). A secondary screening was performed, reviewing the method sections for the remaining 112 studies for analysis-specific requirements. Articles meeting all criteria were retained for final review. From each article, information was extracted pertaining to publication, acquisition details for the dataset(s) examined, demographics, clinical features, preprocessing, implementation of ICA, statistical approach, the results of analyses investigating associations between clinical variables and functional connectivity, and the results of case–control functional connectivity comparisons. To more accurately compare the results across studies within a unified framework, the results of the functional connectivity analyses were translated into the 14 subdomains from the NeuroMark 2.2 template (Jensen et al., 2024b) based on ROI descriptions provided within each article.^1^ The assignment of each ROI to a corresponding NeuroMark subdomain was done manually by visually inspecting spatial maps, entering peak coordinates into Neurosynth (Yarkoni et al., 2011),^2^ and considering the anatomical labels and descriptions provided in the article. Following an initial assignment by first-author KJ, 5% of the ROIs were randomly selected for review and label assignment by senior-author AI following the same procedure. The two expert raters were in 100% agreement. Therefore, despite the possibility of bias which is inherent in the process of network labeling (see Uddin et al., 2023), the categorical assignments into these 14 subdomains were adapted for the purposes of the current literature review.

### Synthesis, relevance, and risk of Bias

The results of case–control functional connectivity comparisons were recorded for each pair of the 14 functional subdomains [e.g., Cerebellar (CB)-Occipitotemporal (OT)]. Specifically, the directionality of case–control group differences was recorded with +1 representing hyperconnectivity, 0 representing no significant difference, −1 representing hypoconnectivity, and NA used to indicate that the given comparisons were not made.^3^ Definitions of dysconnectivity differ across studies, however, to harmonize and aid in interpretation of the results in the current review, hyperconnectivity was defined as an increase in positive directionality (or a decrease in negative directionality) of FC in schizophrenia/psychosis relative to controls and hypoconnectivity was defined as an increase in negative directionality (or a decrease in positive directionality) of FC in schizophrenia/psychosis relative to controls. Static FC was used when available, but dynamic FC was used as an alternative if static FC was not available. When dynamic FC was used, results were recorded for all reported states and directionality was reported according to the same system if all states were in agreement, or based on the directionality of the sum of all states if they differed. A similar winner-take-all approach to recording was applied if the results of multiple datasets were reported separately or if the results within a given subdomain were mixed (e.g., some Cerebellar-Subcortical FC pairs are hyperconnected, while some are hypoconnected), in which case the directionality of significant results were tallied, and the result of the majority was recorded.

Considering the varying degree of similarity between ROIs across studies and the intrinsic connectivity networks (ICNs) defined in the NeuroMark atlas, and also the varying levels of detail articles include when describing ROIs, each study was evaluated to determine the relevance of its results when interpreted within this framework (see Table 1). Five questions were developed to assess the level of detail provided in describing ROIs (i.e., peak coordinates, spatial maps, anatomical labels), the comparability of the ROIs (e.g., *a priori* seed v. ICA-derived), and whether the functional connectivity analyses were comprehensive of the whole brain (e.g., comparisons between all pairs of ROIs). If the relevance was lower, then the study results may be less likely to accurately translate, making them less comparable to other studies in the current review. Responses to each were recorded as “Yes” (1) or “No/Unclear” (0). The overall relevance of the findings for each study were described by the sum of all items, with the possible total ranging from 0 to 5, with higher scores indicating that more criteria were met and therefore had higher relevance to the current review. For ease of interpretation, each study’s relevance was categorized as “High relevance” (score of 5), “Moderate Relevance” (scores ranging from 3 to 4), or “Low Relevance” (scores ranging from 0 to 2).

**Table 1:** Relevance assessment.

1. Were peak coordinates provided for ROI/networks? 2. Were spatial maps plotted for ROI/networks? 3. Were anatomical descriptions/labels provided for ROI/networks? 4. Were all ROI/networks derived through ICA (e.g., IC × IC)? 5. Were all pairs of cortical (C), subcortical (SC), and cerebellar (CB) structures reflected in the ROI/Networks included in the analysis (e.g., C × SC, C × CB, C × C)?

Criteria	Q1	Q2	Q3	Q4	Q5	Overall assessment
Cai et al. (2024)	1	0	1	0	0	Low relevance
Du et al. (2020)	1	1	1	1	1	High relevance
Forlim et al. (2020)	1	1	1	0	0	Moderate relevance
Gong et al. (2019)	1	1	1	0	0	Moderate relevance
Iraji et al. (2024)	0	1	0	1	1	Moderate relevance
Jensen et al. (2024a)	1	1	1	1	1	High relevance
Kwak et al. (2021)	0	1	1	1	0	Moderate relevance
Rong et al. (2023)	1	1	1	0	0	Moderate relevance
Yan et al. (2024)	1	1	1	1	1	High relevance
Zarghami et al. (2023)	1	1	1	1	1	High relevance

The relevance of each study included in the review was rated with the following criteria evaluating the attributes which enable study results to be accurately translated into the NeuroMark 2.2 reference space. If the relevance is lower, then the study results may be less likely to accurately translate, making them less comparable to other studies in the current review. The responses were recorded as follows: Yes = 1, No/Unclear = 0. The overall relevance of each study was determined based on the sum of all items: High Relevance = 5, Moderate Relevance = 3–4, Low Relevance = 0–2.

In addition, an optimized rating system was developed to assess risk of bias for each article in the current review (see Table 2). The JBI critical appraisal checklist for case control studies,^4^ as well as risk of bias measures used in previous literature reviews (Ailion et al., 2017; Aleksonis and King, 2023; Steinberg and King, 2024) were referenced in developing an optimized rating system to assess each article identified for the current review. Thirteen questions were developed regarding study recruitment and inclusion criteria, the description, definition, and comparability of case/control samples, data acquisition and preprocessing, confounds addressed, and whether they corrected for multiple comparisons. Responses to each were recorded as “Yes” (1) or “No/Unclear” (0). For studies using datasets reported in previous studies (e.g., FBIRN) information provided previously (e.g., description of imaging acquisition) was considered. The overall bias assessment of each study was determined based on the sum of all items, with totals ranging from 0 to 13, with lower scores indicating that fewer criteria were met and therefore had a higher risk of bias. For ease of interpretation, each study’s risk of bias was categorized as “Low Risk” (scores ranging from 11 to 13), “Moderate Risk” (scores ranging from 7 to 10), or “High Risk” (scores ranging from 0 to 6).

**Table 2:** Risk of bias assessment.

Were the inclusion criteria clearly described? Was the sample clearly described (demographics and recruitment)? Were case/control groups well-defined (screening and Dx)? Were case/control groups matched? Were case/control samples sufficiently large (N ≥30)? Were acquisition methods clearly described? Was the preprocessing clearly described? Did they correct for head motion (preprocessing and accounted for in analysis)? Did they correct for age? Did they correct for sex? Did they correct for race (if applicable/racially diverse sample)? Did they correct for multiple imaging sites (if applicable)? Did the analysis correct for multiple comparisons?

Criteria	Q1	Q2	Q3	Q4	Q5	Q6	Q7	Q8	Q9	Q10	Q11	Q12	Q13	Overall assessment
Cai et al. (2024)	1	1	1	1	1	1	1	1	1	1	0	1	1	Low risk
Du et al. (2020)	1	1	1	1	1	1	1	1	1	1	0	1	1	Low risk
Forlim et al. (2020)	1	1	1	1	1	1	1	1	1	1	0	1	1	Low risk
Gong et al. (2019)	1	1	1	1	1	0	1	1	1	1	0	1	1	Low risk
Iraji et al. (2024)	1	1	1	1	1	1	1	1	1	1	0	1	0	Low risk
Jensen et al. (2024a)	1	1	1	1	1	0	1	1	1	1	1	1	1	Low risk
Kwak et al. (2021)	1	1	1	1	1	1	1	1	1	1	0	1	1	Low risk
Rong et al. (2023)	1	1	1	1	1	1	1	1	1	1	1	1	1	Low risk
Yan et al. (2024)	1	1	1	0	1	1	1	1	1	1	0	1	0	Moderate risk
Zarghami et al. (2023)	1	1	1	1	1	1	1	1	1	1	0	1	1	Low risk

The risk of bias for each study was rated with the following criteria. The responses were recorded as follows: Yes = 1, No/Unclear = 0. For studies using datasets reported in previous studies (e.g., FBIRN), information provided in the original study was considered. The overall bias assessment of each study was determined based on the sum of all items: Low Risk = 11–13, Moderate Risk = 7–10, High Risk = 0–6.

## Results

### Study selection and sample characteristics

**Figure 2:**
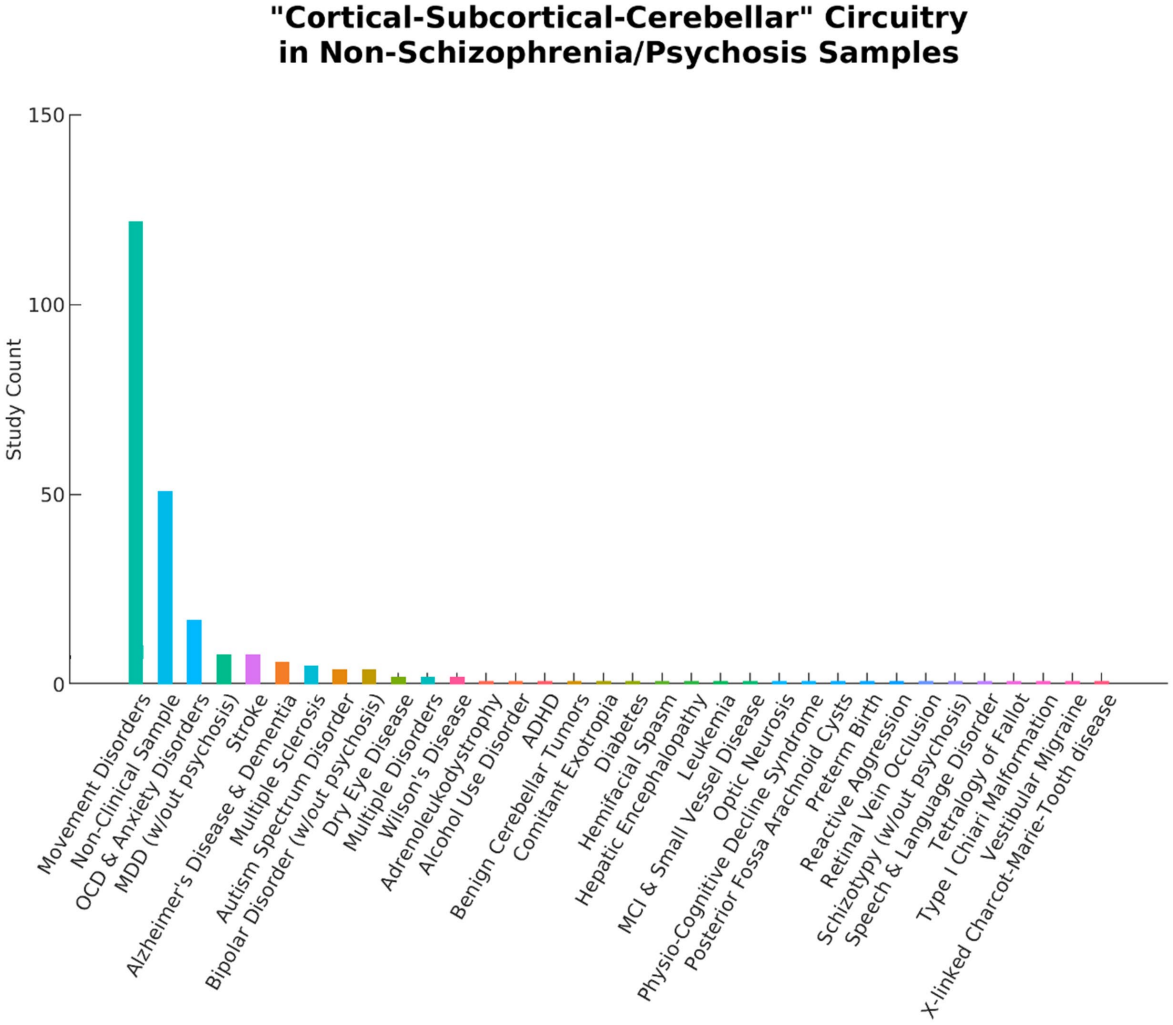
A total of 253 articles using the term “Cortical–Subcortical-Cerebellar” to describe their findings were excluded from the current literature review because they did not include schizophrenia or psychosis samples. The bar chart above displays a wide range of study samples and the number of articles for each. Movement disorders include Parkinson’s Disease, Tourette’s syndrome, tremors, dystonia, chorea, ataxia, myoclonus, epilepsy, and lateral sclerosis. OCD, Obsessive Compulsive Disorder; MDD, Major Depressive Disorder; ADHD, Attention Deficit and Hyperactivity Disorder; MCI, Mild Cognitive Impairment.

Initial searches on PubMed, APA PsychInfo, and Google Scholar identified 683 records. After screening, 10 articles were determined to be eligible for inclusion in the final review. The reasons for exclusion are reported in Figure 1. Notably, 253 articles were excluded because they did not include a schizophrenia or psychosis sample (see Figure 2). Even within the final selection of articles, the sample types varied across studies (see Table 3), with two studies examining transdiagnostic early psychosis samples (Jensen et al., 2024a; Kwak et al., 2021), one study investigating comparisons with an early-onset schizophrenia sample (Cai et al., 2024), and a majority of seven studies investigating comparisons with chronic schizophrenia. Six studies utilized a single dataset, one study utilized two datasets but reported analyses of each separately (Du et al., 2020), one study utilized two datasets combined into one for its analyses (Jensen et al., 2024a), one study utilized three datasets combined (Iraji et al., 2024), and one study utilized five datasets combined (Yan et al., 2024). Four studies utilized overlapping datasets, each with unique combinations of COBRE (Iraji et al., 2024; Yan et al., 2024; Zarghami et al., 2023), FBIRN (Du et al., 2020; Iraji et al., 2024; Yan et al., 2024), and MPRC (Du et al., 2020; Iraji et al., 2024; Yan et al., 2024). The sample sizes ranged from 76 (35 schizophrenia)—2,615 (1,302 schizophrenia). Cai et al. (2024) utilized an early-onset schizophrenia sample with a mean age of 15. The early psychosis studies examined slightly older samples with a mean age around 23 (Jensen et al., 2024a; Kwak et al., 2021). Rong et al. (2023) also utilized a relatively young chronic schizophrenia sample with a mean age of 25. The remaining six studies examined chronic schizophrenia samples with mean ages ranging from 35 to 39.

**Table 3:** Demographic characteristics of the studies included in the review.

Study	Dataset(s)	Sample type	Total N	Case N (male/female)	Control N (male/female)	Case age^j^ mean ± SD	Control age mean ± SD
Cai et al. (2024)	West China Hospital Sichuan University	Early-Onset SZ^f^	160	97 (34/63)	63 (27/36)	15.1 ± 1.6	14.3 ± 2.9
Du et al. (2020)	FBIRN^a^; MPRC^b^	Chronic SZ	281; 388^i^	137 (103/34); 150 (98/52)	144 (104/40); 238 (94/144)	39.0 ± 11.4; 38.7 ± 14.1	37.2 ± 11.0; 40.2 ± 15.2
Forlim et al. (2020)	St. Hedwig Hospital (Erlangen, Germany)	Chronic SZ	76	35 (21/14)	41 (24/17)	35.3 ± 10.8	35.2 ± 11.0
Gong et al. (2019)	University of Electronic Science and Technology of China	Chronic SZ	96	54 (34/20)	42 (24/18)	38.1 ± 12.6	39.6 ± 11.8
Iraji et al. (2024)	FBIRN; COBRE^c^; MPRC	Chronic SZ	508	193 (154/39)	315 (185/130)	38.6 ± 13.3	38.4 ± 12.7
Jensen et al. (2024a)	University of Pittsburgh; Johns Hopkins	Early psychosis^g^	247	117 (81/36)	130 (71/59)	23.2 ± 4.4	23.3 ± 4.0
Kwak et al. (2021)	Seoul National University	Early psychosis^h^	80	40 (18/22)	40 (20/20)	22.9 ± 5.6	22.6 ± 3.9
Rong et al. (2023)	Wuhan University	Chronic SZ	365	196 (98/98)	169 (85/84)	25.4 ± 5.6	25.0 ± 4.9
Yan et al. (2024)	BSNIP^d^; COBRE; FBIRN; MPRC; 754 Chinese Han sample (7 sites)^e^	Chronic SZ	2,615	1,302 (815/487)	1,313 (634/679)	35.8 ± 12.6	34.2 ± 11.7
Zarghami et al. (2023)	COBRE	Chronic SZ	140	66 (53/13)	74 (51/23)	38.3 ± 14.2	35.8 ± 11.6

^a^FBIRN: Function Biomedical Informatics Research Network data repository (Keator et al., 2016). ^b^MPRC: Maryland Psychiatric Research Center (Adhikari et al., 2019). ^c^COBRE: Center for Biomedical Research Excellence (Aine et al., 2017). ^d^BSNIP: Bipolar-Schizophrenia Network on Intermediate Phenotypes (Tamminga et al., 2013). ^e^754-Chinese Han Sample (7 sites): Peking University Sixth Hospital, Beijing Huilongguan Hospital, XinXiang Hospital Simens, Xinxiang Hospital GE, Xijing Hospital, Renmin Hospital of Wuhan University, Zhumadian Psychiatric Hospital (Yan et al., 2019). ^f^The early-onset schizophrenia sample consists of individuals diagnosed with schizophrenia before age 18. ^g^The early psychosis sample is transdiagnostic including individuals with schizophrenia, schizoaffective disorder, schizophreniform, bipolar disorder, major depressive disorder, and others within 2 years of first psychosis onset. ^h^The early psychosis sample is transdiagnostic including individuals with schizophrenia, schizophreniform, and schizoaffective disorder within 2 years of first psychosis onset. ^i^Demographics are reported separately for the two datasets in Du et al. (2020) because the analyses and results are reported for each dataset separately. ^j^Age is reported in years. SZ, schizophrenia.

### Clinical characteristics and associations with FC

Clinical characteristics are summarized in Table 4. The clinical samples in all studies were diagnosed using DSM-IV or DSM-IV-TR criteria, except for the sample in Forlim et al. (2020) which was diagnosed based on ICD-10 criteria. Six of the studies utilized the positive and negative syndrome scale (PANSS; Kay et al., 1987) for schizophrenia, with four of them reporting associations between symptom severity and FC (Cai et al., 2024; Du et al., 2020; Gong et al., 2019; Rong et al., 2023). However, these findings were mixed, with no consistent patterns (see Supplementary Appendix 1). The early-onset schizophrenia sample in Cai et al. (2024) had relatively high symptom severity with a mean PANSS total of 78.5 and was also characterized by a relatively short duration of illness (DOI < 1 year) and lower antipsychotic use (average chlorpromazine equivalence of 140.7 milligrams per day). Symptom severity was not as severe in the early psychosis samples (Jensen et al., 2024a; Kwak et al., 2021), and both had a much shorter DOI than the chronic schizophrenia samples. The sample reported in Jensen et al. (2024a) also had relatively low average antipsychotic use.

**Table 4:** Characteristics of the clinical samples included in the review.

Study	Diagnostic criteria	Symptom scale	Symptom score mean ± SD	DOI^d^ mean ± SD	CPZ^e^ mean ± SD	Findings related to symptom severity, DOI, CPZ, and cognitive impairment^h^
Cai et al. (2024)	DSM-IV	PANSS	78.5 ± 24 PANSS total	8.7 ± 13.2 months	140.7 ± 210.5^f^	DM-ET FC positively correlated w/PANSS general; SOC positively correlated with FR-ET FC (did not survive FDR)
Du et al. (2020)	DSM-IV-TR; DSM-IV	PANSS	–	–	–	CB-ET FC negatively correlated w/PANSS negative
Forlim et al. (2020)	ICD-10	SANS; SAPS	~13.9^a^ PANSS negative; ~15.1 PANSS positive	9.4 ± 8.8 years	317.1 ± 221.6	DM-DM FC negatively correlated w/SANS composite and apathy; CPZ did not correlate w/DM-DM FC, it also had no impact as a covariate
Gong et al. (2019)	DSM-IV	PANSS	~63.2^b^ PANSS total	13.9 ± 10.6 years	281.6 ± 122.7	ET-SM FC positively correlated w/PANSS total, PANSS general, and PANSS negative; ET-IT and ET-OT FC positively correlated w/PANSS negative; ET-ET FC negatively correlated w/PANSS negative; ET-ET FC negatively correlated w/DOI; CPZ negatively correlated w/ET-CB FC and positively correlated w/OT-ET, SM-ET FC
Iraji et al. (2024)	DSM-IV-TR; DSM-IV-TR; DSM-IV	PANSS	61.1 ± 15.4 PANSS total	–	344.8 ± 274.1	CPZ did not correlate w/IT/TP-SM, SA-DM FC
Jensen et al. (2024a)	DSM-IV	SANS; SAPS	~16^c^ PANSS negative; ~15 PANSS positive	11 (median) months	210.5 ± 220.9	Nothing survived FDR; SANS positively correlated w/BG-FR FC, SM-EH FC; SANS negatively correlated w/IT-OT FC, IT-FR FC, OC-FR FC, CB-CB FC; SAPS positively correlated w/BG-FR FC; SAPS negatively correlated w/ET-CB FC, EH-CB FC
Kwak et al. (2021)	DSM-IV	PANSS	69.0 ± 14.3 PANSS total	5.7 ± 3.8 months	378.5 ± 296.0^g^	–
Rong et al. (2023)	DSM-IV	PANSS; SANS; SAPS	82.7 ± 11.6 PANSS total	46.2 ± 54.1 months	373.8 ± 283.7	Nothing survived FDR; FR × SM FC positively correlated w/PANSS total and negative; DM × DM FC negatively correlated w/PANSS general; FR × SM FC positively correlated w/PANSS positive; IT-SM FC negatively correlated with DOI; CPZ positively correlated w/TP-SM FC
Yan et al. (2024)	DSM-IV; DSM-IV-TR; DSM-IV-TR; DSM-IV; DSM-IV-TR	–	–	–	–	–
Zarghami et al. (2023)	DSM-IV-TR	–	–	–	363.1 ± 305	All 7 MATRICS domains showed significant impairments in SZ; CCA top results were SM-SM and PL-TP EF, top MCCB traits were social cognition, reasoning/problem solving, working memory

^a^SANS and SAPS composite scores have been converted to PANSS Negative and Positive scores to make comparisons easier across studies (see van Erp et al., 2014).^b^The mean PANSS total is not provided, however, it has been estimated as the sum of mean positive, negative, and general subscales (provided). ^c^SANS and SAPS global scores have been converted to PANSS Negative and Positive scores to make comparisons easier across studies. ^d^DOI: Duration of illness. ^e^CPZ: Chlorpromazine equivalence in milligrams per day. ^f,g^The olanzapine dose reported has been converted to CPZ based on Leucht et al. (2015). ^h^DM, Default Mode subdomain, ET, Extended Thalamic subdomain, FC, Functional Connectivity, CB, Cerebellar domain, SM, Sensorimotor domain, IT, Insular-Temporal subdomain, BG, Basal Ganglia subdomain, FR, Frontal subdomain, EH, Extended Hippocampal subdomain, OT, Occipitotemporal subdomain, OC, Occipital subdomain, TP, Temporoparietal subdomain, SA, Salience subdomain, PL, Paralimbic subdomain, SOC, Stockings of Cambridge from Cambridge Neuropsychological Test Automated Battery. SZ, schizophrenia.

### Case–control group differences in FC

**Figure 3:**
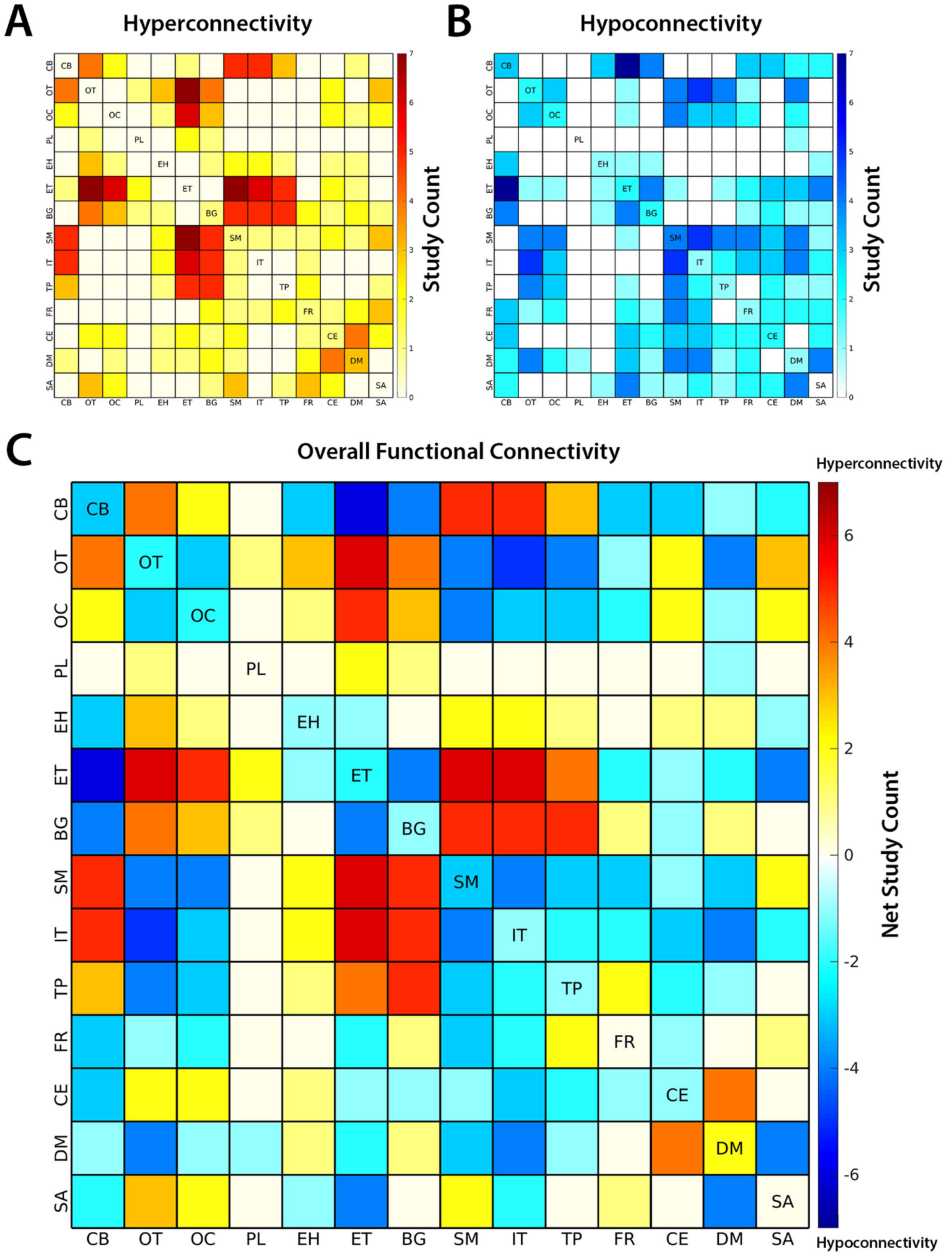
The number of studies reporting **(A)** hyperconnectivity and **(B)** hypoconnectivity between functional subdomains of the brain are shown above. Hyperconnectivity represents an increase in the positive directionality of functional connectivity (FC) in schizophrenia (SZ) relative to controls and hypoconnectivity represents a relative decrease (or increase in negative directionality) in SZ. **(C)** The overall patterns of FC are represented by net study count, where studies reporting hyperconnectivity are assigned +1 and studies reporting hypoconnectivity are assigned −1. The 14 subdomains are based on the NeuroMark 2.2 multi-scale template: cerebellar (CB), visual-occipitotemporal (OT), visual-occipital (OC), paralimbic (PL), subcortical-extended hippocampal (EH), subcortical-extended thalamic (ET), subcortical-basal ganglia (BG), sensorimotor (SM), higher cognition-insular temporal (IT), higher cognition-temporoparietal (TP), higher cognition-frontal (FR), triple network-central executive (CE), triple network-default mode (DM), and triple network-salience (SA).

**Figure 4:**
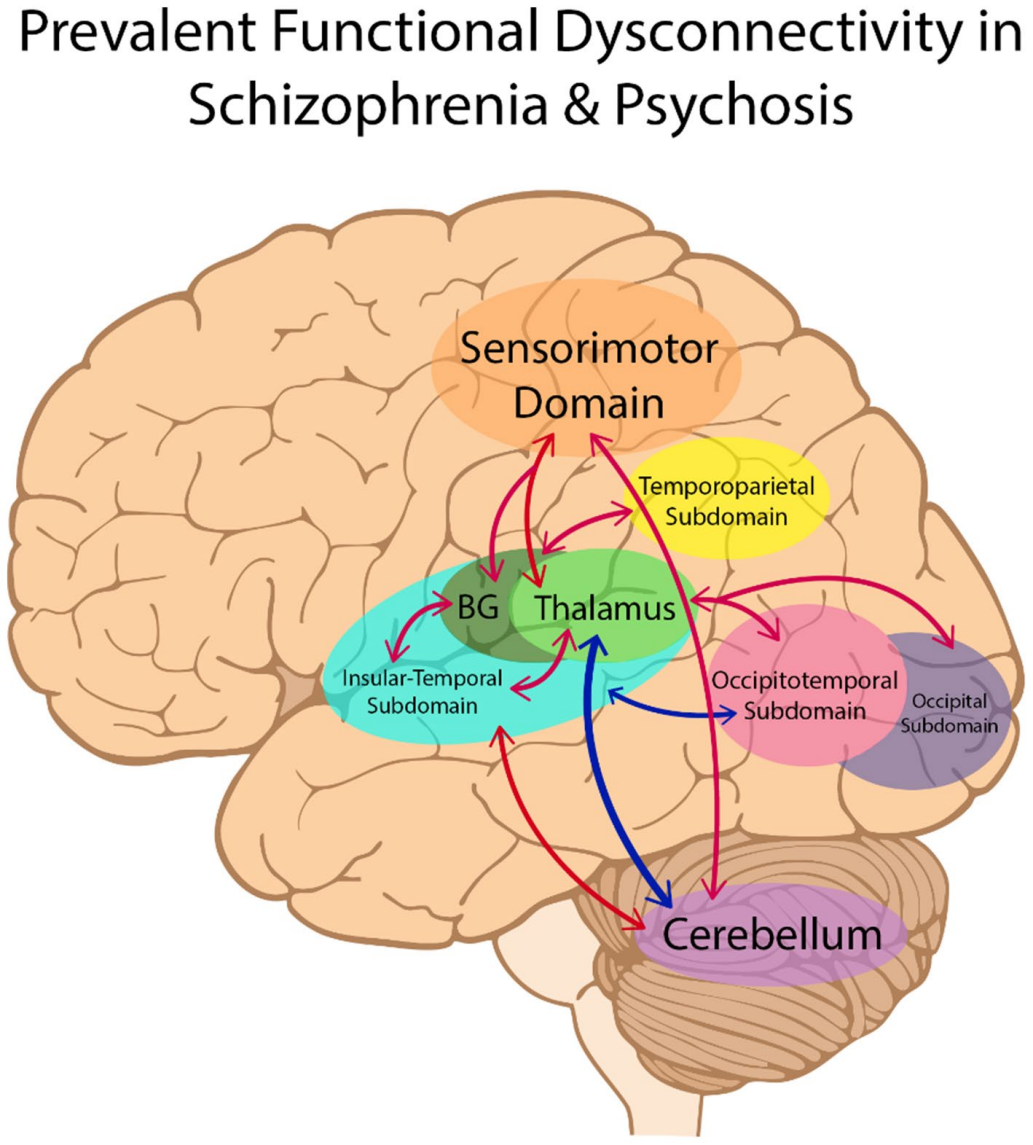
The most consistently reported patterns of dysconnectivity (with a net study count of 5 or more) are portrayed above. Hyperconnectivity in schizophrenia relative to controls is represented by red arrows between brain regions, with hypoconnectivity represented in blue. BG, Basal Ganglia.

The results of case–control functional connectivity for each pair of the 14 functional subdomains is summarized in Figures 3, 4. Figure 3A displays the number of studies reporting hyperconnectivity across NeuroMark subdomains in schizophrenia/psychosis relative to controls, Figure 3B displays the number of studies reporting hypoconnectivity, and Figure 3C displays the net study count with hyperconnectivity represented as +1 and hypoconnectivity represented as −1. The most consistently reported patterns of dysconnectivity (net study count ≥ 5) are portrayed in Figure 4, with red arrows representing hyperconnectivity in schizophrenia/psychosis relative to controls and blue arrows representing hypoconnectivity. Although many notable patterns were observed, the most prevalent were cerebellar-cortical [CB-Sensorimotor (SM) and CB-Insular-Temporal (IT)] hyperconnectivity, cerebellar-subcortical [CB-Extended Thalamic (ET)] hypoconnectivity, subcortical–cortical [ET-SM, ET-OT, ET-Occipital (OC), ET-IT, Basal Ganglia (BG)-SM, BG-Temporoparietal (TP), and BG-IT] hyperconnectivity, and cortico-cortical (IT-OT) hypoconnectivity.

### Consistency, risk of bias, and certainty of evidence

**Figure 5:**
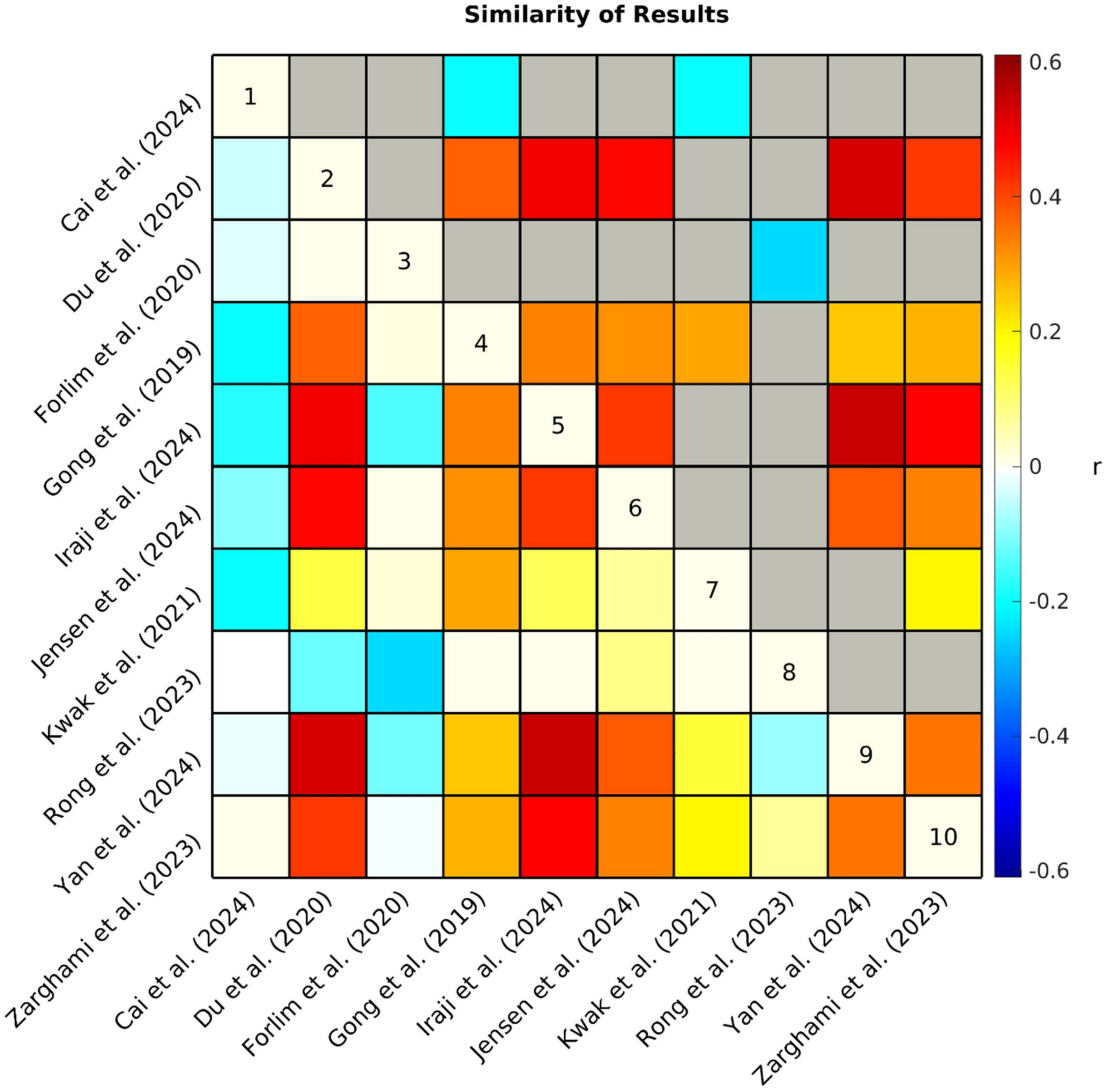
Each study produced an array of values (i.e., +1, 0, −1, NA) representing hyper/hypoconnectivity between case/control groups for each pair of subdomains. The similarity (Pearson correlation coefficient *r*) of observed patterns of functional connectivity across studies is represented by the correlation matrix above. Non-significant (*p* > 0.05) associations have been grayed out in the upper triangle. NA values were interpreted as zero, therefore, a lower correlation suggests that studies had less similar results either because the observed patterns of connectivity differed between studies or because of differences in analytical approach (e.g., fewer comparisons were made due to differences in network definitions).

To further evaluate the consistency of the FC case–control comparison results across studies, a Pearson correlation between the extracted FC results was calculated between studies (see Figure 5). While seven of the 10 studies demonstrated relatively high similarity of results, Rong et al. (2023) displayed weaker correlations and Cai et al. (2024) and Forlim et al. (2020) appeared to be weakly anticorrelated with the common patterns.

Nine of the 10 studies were assessed as having low risk of bias, demonstrating that in general samples were carefully considered and appropriately matched, acquisition and preprocessing were clearly described, applicable confounds were controlled for, and analyses corrected for multiple comparisons. One notable exception is that only two studies (Jensen et al., 2024a; Rong et al., 2023) took race and ethnicity into consideration in their analyses. One study (Yan et al., 2024) was assessed as having moderate risk of bias, because they did not correct for multiple comparisons as only schizophrenia minus control FC differences were provided (i.e., no test statistics were provided as these results were only supplementary to the primary analyses). In addition, it appears that the schizophrenia and control samples in Yan et al. (2024) were not matched by sex, although this was controlled for in the analysis.

The studies varied considerably in their implementation of ICA (see Table 5). The ROIs in three of the studies (Du et al., 2020; Jensen et al., 2024a; Yan et al., 2024) represented ICNs delineated through ICA spatially constrained to the NeuroMark 1.0 template (Du et al., 2020) which includes 53 optimized ICNs selected from a model order of 100. Although not identical, the ROIs utilized in Zarghami et al. (2023) were very similar, delineated through ICA spatially constrained to a 50 ICN template from Allen et al. (2014) which also used a model order of 100. Notably, these four studies were assessed as highly relevant (see Table 1) to the current review because they were evaluated to more accurately translate into the NeuroMark 2.2 reference space due to attributes of how the networks were delineated and described. Iraji et al. (2024) utilized the Group ICA of fMRI Toolbox (GIFT)^5^ to perform group-level spatially-constrained ICA similar to the previous four studies, however, they utilized only 14 ICNs selected from a model order of 20. Lower model orders of ICA tend to be less granular which may limit the study’s ability to isolate effects and translate into the NeuroMark reference space which incorporates higher model orders (see Abou-Elseoud et al., 2010; Mirzaeian et al., 2025). Furthermore, this study was assessed as having moderate relevance, because the ICNs were described with limited detail, lowering the confidence in the accuracy of their assignment into NeuroMark 2.2 subdomains.

**Table 5:** Notes about the implementation of independent component analysis (ICA) in the functional connectivity analyses from each study included in the review.

Study	Notes on implementation of ICA	Model order	Final ICNs	Labeling
Cai et al. (2024)	ICA used to parcellate thalamus; FC represents thalamic subdivisions by whole-brain voxel-level analysis	–	5 thalamic ROIs, whole-brain voxel-level	“Yeo Network” and another not specified for labeling “Regions”
Du et al. (2020)	ICA spatially-constrained to NeuroMark 1.0	100	53	NeuroMark 1.0
Forlim et al. (2020)	Used GIFT/ICA to extract ICNs, visually inspected and labeled ICNs, then compared within network connectivity between case/control; did not examine between network FC and did not include CB ICNs	21	main analysis included DMN only, but exploratory included additional 6 ICNs	“Templates in GIFT” and “experts”
Gong et al. (2019)	ICA used to parcellate thalamus; FC represents thalamic subdivisions by whole-brain voxel-level analysis	–	6 thalamic ROIs, whole-brain voxel-level	–
Iraji et al. (2024)	Used GIFT/ICA to extract ICNs, visually inspected and labeled 14 ICNs, 4 states of dynamic FNC reported between all 14 ICNs	20	14	Previous knowledge/studies
Jensen et al. (2024a)	ICA spatially-constrained to NeuroMark 1.0	100	53	NeuroMark 1.0
Kwak et al. (2021)	ICA used on thalamus by whole-brain seed-based correlation maps to identify 20 thalamus-related ICNs, FC between thalamus and network ROIs	20	20	Based on networks reported by Yuan et al. (2016)
Rong et al. (2023)	HCP ICA templates, 32 ROIs (avg of all voxels within seed) from 8 ICA derived RSNs, FC between 32×32 ROIs, no subcortical/thalamic ROIs	–	ICA templates used as mask for ROIs	HCP ICA templates
Yan et al. (2024)	ICA spatially-constrained to NeuroMark 1.0, reported difference between SZ-control (but no t-stats/*p*-values)	100	53	NeuroMark 1.0
Zarghami et al. (2023)	ICA spatially-constrained to Allen et al. (2014) 50 ICN template	100	50	Allen et al. (2014)

SZ, schizophrenia.

Forlim et al. (2020) differed more in their approach, using GIFT to perform blind ICA with a model order of 21. They also differed in that they selected only a single ICN to represent the DMN, which was the focus of their primary analyses (i.e., DMN-DMN FC). They also performed limited exploratory analyses with an additional six ICNs they selected from the 21. In both primary and exploratory analyses, however, the ICNs were used to perform cluster-based analyses which differed from the whole-brain FC comparisons made in the previous five studies which compared the correlation between the time courses of other ICNs. This study was assessed as having moderate relevance to the current review due to these key differences in analytical approach. Rong et al. (2023) also differed in that it used ICA-derived templates to select 32 ROIs. The FC from these ROIs was calculated from the average of all voxels within each seed rather than correlations between ICN time courses. Furthermore, they did not include any subcortical ROIs in their analyses and were assessed as having only moderate relevance to the current review. The remaining three studies (Cai et al., 2024; Gong et al., 2019; Kwak et al., 2021) differed considerably from the previously mentioned studies in that ICA was performed only on the thalamus. Specifically, Kwak et al. (2021) identified 20 thalamus-related network ROIs spanning across the brain and examined seed-based functional connectivity between them. The relevance of this study was limited (assessed as moderate) due to a limited description of these networks as well as limited comparisons made between these networks. Cai et al. (2024) and Gong et al. (2019) used ICA to parcellate the thalamus into five and six thalamic ROIs, respectively. In both studies, FC represented the correlation between thalamic subdivisions and whole-brain voxel-level analyses. Due to this limitation, Gong et al. (2019) was assessed as having only moderate relevance. Cai et al. (2024) was further limited to low relevance as spatial maps were not provided for all the relevant ROIs, making it more difficult to accurately translate the results into the NeuroMark reference space.

## Discussion

### Cortical–subcortical-cerebellar terminology

Many articles in the initial search were excluded because they did not include a schizophrenia or psychosis sample (see Figure 2). Movement disorders were the largest contributor, composing 122 of these articles. Interestingly, the initial search identified nearly as many studies examining samples with movement disorders as those examining schizophrenia or psychosis. It is possible that this finding may be due to overlapping affected brain circuitry between schizophrenia and movement disorders (Andreasen et al., 1998). Indeed, some of the most prevalent patterns reported in the current review were dysconnectivity in motor pathways (see Figure 4). Regardless of the reason, it is apparent that not only is the term “cortical–subcortical-cerebellar” frequently used to describe a prominent category of pathology unrelated to schizophrenia and psychosis, but it is also used to describe a wide range of disorders and even non-clinical samples. This undermines the usefulness of the term for characterizing the biological profiles of schizophrenia and psychosis and further demonstrates the need for more precise language in defining the key alterations in rs-fMRI in individuals with these clinical profiles.

### Aberrant connectivity in schizophrenia and psychosis

The primary objective of the current review was to delineate and describe in greater detail the most prevalent patterns of dysconnectivity observed in schizophrenia and psychosis with data-driven ICA approaches. The most prominent patterns observed in schizophrenia and psychosis can be summarized as hypoconnectivity between cerebellar and subcortical structures, specifically between the cerebellum and thalamus, with hyperconnectivity between cerebellar and cortical structures (SM and IT) as well as between subcortical (ET and BG) and cortical structures (SM, TP, IT, OT, and OC). Cortical–cortical connectivity (e.g., IT-OT) was less commonly reported, but when it was the patterns typically reflected hypoconnectivity in schizophrenia and psychosis.

Consistent with Andreasen et al. (1998) theory of cognitive dysmetria and the cerebello-thalamo-cortical framework (Harikumar et al., 2023; Hwang et al., 2022), the cerebellum and thalamus appear to be key nodes, and disruptions in circuitry between them and the cortex appear to be characteristic of schizophrenia and psychosis. Indeed, cerebellocortical and thalamocortical dysconnectivity have been suggested as a substrate of under-regulated cognitive processes (Andreasen et al., 1999; Clark et al., 2020). Notably, the most consistent patterns of cortical dysconnectivity were centered in regions associated with primary and secondary sensory and motor processing. Specifically, the SM spans across primary somatosensory and motor cortex as well as related heteromodal association areas (Jensen et al., 2024b). Motor-related symptoms are often experienced in schizophrenia and disruptions in sensorimotor circuitry are widely implicated across studies (Andreasen et al., 1998; Cattarinussi et al., 2023; Walther et al., 2017). Neurological abnormalities in sensorimotor performance are sometimes referred to as neurological soft signs and have been suggested to be present during the early stages of schizophrenia and psychosis and potentially even pre-date illness onset (Dazzan and Murray, 2002). Although sensorimotor symptoms and alterations in sensorimotor FC are frequently attributed to the effects of antipsychotic medication targeting the dopamine system (Kraguljac et al., 2013; Lerner and Miodownik, 2011; Sarpal et al., 2015; Yu et al., 2021), there is evidence that the relationship may be more complex as sensorimotor symptoms and alterations in SM connectivity have been observed in drug-naïve schizophrenia, suggesting that they may also be independently related to the pathophysiology of schizophrenia (Zhang et al., 2019). The reviewed studies further demonstrate how antipsychotic treatment may confuse results depending on characteristics of the sample. For example, Cai et al. (2024) reported ET-SM hypoconnectivity in contrast to the ET-SM hyperconnectivity reported by the majority. Importantly, the sample in Cai et al. (2024) had the lowest antipsychotic dosage as well as a relatively short DOI. That being said, Jensen et al. (2024a) reported ET-SM hyperconnectivity in a sample with only a small increase in dosage and duration. Unfortunately, only four of the reviewed studies specifically tested for associations between medication and FC, with two implicating SM circuitry (Gong et al., 2019; Rong et al., 2023) and two reporting null results (Forlim et al., 2020; Iraji et al., 2024; see Supplementary Appendix 1). Consistent with these findings, Gong et al. (2019) and Rong et al. (2023) also identified associations between SM circuitry and DOI (Supplementary Appendix 1), which is generally associated with increased medication use. Further investigation, such as studies employing analyses stratified by dosage and illness duration, are warranted to disentangle the specific effects of medication and chronicity on sensorimotor symptoms and processes.

Sensory association areas within the SM, along with the IT and TP, encompass key cortical regions implicated in auditory and language networks. Specifically, primary auditory cortex (A1) is located within the medial superior temporal lobe (a region with high spatial overlap with the IT) and the dorsal auditory stream leads from A1 to the parietal lobe (overlapping with the TP and SM) (Jensen et al., 2024b; Purves et al., 2001; Rauschecker and Tian, 2000). Prior studies have attributed auditory verbal hallucinations (AVHs) to disruptions in the processing of auditory information across these networks (Jardri et al., 2011; Kompus et al., 2011; Kuhn and Gallinat, 2012). Similarly, the OT and OC encompass cortical regions primarily associated with visual sensory processing, including the primary visual cortex (V1), visual association cortex (V2–V5), and the ventral visual stream (Goodale and Milner, 1992; Huff et al., 2024; Jensen et al., 2024b). Like AVHs and auditory cortex, visual hallucinations (VHs) have been attributed to disruptions in processing in visual cortex, although the underlying mechanisms remain unclear (Collerton et al., 2023).

### Addressing heterogenous FC across studies

There was a consensus in many of the findings across studies and although some of this may be driven partly by similarities in datasets and analytical approach, these factors cannot fully account for the consistent findings, as there were also many differences. For example, among the seven studies showing higher similarity of results, three of them included subjects from the FBIRN and MPRC datasets (Du et al., 2020; Iraji et al., 2024; Yan et al., 2024) and three included subjects from the COBRE dataset (Iraji et al., 2024; Yan et al., 2024; Zarghami et al., 2023), however, different combinations of these datasets enabled all of these studies to examine FC group differences in unique samples. Furthermore, Gong et al. (2019), Jensen et al. (2024a), and Kwak et al. (2021) demonstrated high similarity with these studies despite their use of completely different datasets. In order to further explore the impact of the sample heterogeneity across studies on the collective trends in reported FC group differences (Figure 3), we performed a sensitivity analysis by removing two studies from our composite study count to eliminate overlap in datasets and achieve greater consistency in sample size across studies (see Supplementary Appendix 2). We found that the overall patterns remained mostly unchanged. On a similar note, Jensen et al. (2024a) and Kwak et al. (2021) both utilized early psychosis samples with mixed diagnostic groups as opposed to the schizophrenia samples utilized by the other eight studies, however, it appears that neither reduced chronicity, nor the inclusion of additional diagnostic groups made a substantial difference, as these two studies reflected relatively high similarity with most of the other studies. Although, further investigation into the effects of chronicity may be warranted when considering the divergent results of the early-onset schizophrenia sample observed in Cai et al. (2024), as well as the associations between DOI and dysconnectivity reported by Gong et al. (2019) and Rong et al. (2023).

While differences in analytical approach likely contributed to differences in results across studies, this cannot completely account for the observed differences in FC and in some cases these methodological differences may have had minimal impact. For example, four of the studies were highly similar in their implementation of ICA (Du et al., 2020; Jensen et al., 2024a; Yan et al., 2024; Zarghami et al., 2023), with three of them defining ROIs as ICNs which were spatially constrained to the same standardized network template (Du et al., 2020; Jensen et al., 2024a; Yan et al., 2024). However, some of the other studies showing high similarity of results had fairly large differences in analytical approach, for example, Iraji et al. (2024) examined dynamic FC in 14 ICNs selected from a model order of 20 but was highly similar to many of the studies utilizing a model order of 100 to examine static FC, with especially high similarity to Yan et al. (2024). Similarly, Kwak et al. (2021) utilized a model order of 20 to examine thalamus by whole-brain FC and showed relatively high similarity with the studies utilizing ICNs from higher model orders. Gong et al. (2019) differed even more, using ICA only in its parcellation of thalamic subdivisions which were used as seeds in a whole-brain voxel-level analysis, yet demonstrated fairly high similarity of results when compared with the six studies using only ICA-derived ICNs as ROIs. Together, these findings suggest that the differences in results were not merely a product of the differences in samples and analytical approach but instead were likely driven by additional factors as well.

One possibility is that the divergent studies may be capturing different biological profiles of schizophrenia. Schizophrenia is likely biologically heterogenous and different samples may present different combinations of biologically different subgroups, displaying mixed results (Andrés-Camazón et al., 2025; Clementz et al., 2016; Feczko et al., 2019). There is evidence for this in the varying clinical profiles of the samples examined in Rong et al. (2023), Cai et al. (2024), and Forlim et al. (2020; see Table 4), the three studies displaying low similarity with the majority (see Figure 5). Specifically, Rong et al. (2023) observed the highest symptom severity among the reviewed samples. Similarly, Cai et al. (2024) examined FC in an early-onset schizophrenia sample displaying higher symptom severity than most of the reviewed samples. Early-onset schizophrenia has previously been reported to have a different clinical profile than adult-onset schizophrenia (Immonen et al., 2017; Remschmidt and Theisen, 2012), which appears to be largely what the other studies are capturing in their samples although their participants are not explicitly described that way. Indeed, early-onset (also referred to as childhood or adolescent onset) schizophrenia has been suggested to reflect a different biological profile as well, with differences reported in genetic associations (Alkelai et al., 2023) and functional connectivity (Zhang et al., 2021). A study examining age-related changes in FC even observed opposite patterns in FC directionality between younger and older psychosis cohorts (Passiatore et al., 2023). Similarly, Anticevic et al. (2015) observed hyperconnectivity in prefrontal cortex regions where hypoconnectivity is more commonly observed and suggested that some patterns of aberrant FC may be inverted in early-course schizophrenia, reflecting dynamic alterations in FC as individuals transition to chronic schizophrenia, possibly resulting from compensatory mechanisms. It is possible that age-related factors such as those described in prior work may help account for the anticorrelated results observed between Cai et al. (2024) and majority of the findings included in the current review. Future studies are needed to substantiate this potential reversal of dysconnectivity between early and adult-onset cohorts.

In contrast to Cai et al. (2024) and Rong et al. (2023), the sample in Forlim et al. (2020) appeared to have relatively low symptom severity, although differences in reported scales makes it difficult to compare across studies (see van Erp et al., 2014). In addition, Forlim et al. (2020) utilized ICD-10 which differs from the diagnostic criteria of the DSM-IV and DSM-IV-TR which was utilized by the other nine studies. Although generally considered to be comparable, it has been suggested that the ICD-10 results in a broader concept of schizophrenia than the DSM-IV (Cheniaux et al., 2009; Lindström et al., 1997). Forlim et al. (2020) also had the smallest sample size with only 35 individuals with schizophrenia. Together, these factors may have resulted in a more unique clinical sample. Although the samples in Rong et al. (2023), Cai et al. (2024), and Forlim et al. (2020) all share the diagnostic label of schizophrenia, each appears to represent a unique clinical profile which may have contributed to its divergent patterns of FC. The field would benefit from approaches which leverage biological measures to further explore the possibility of unique signatures of FC among subtypes within psychosis spectrum disorders such as schizophrenia (Ballem et al., 2025).

The differences in terminology used to describe functional units across studies should also be considered. As previously mentioned, variability in terminology or inconsistencies in how the same terminology is applied to different functional entities (Uddin et al., 2023), as well as individual variability in their spatial maps (Jensen et al., 2024b) may potentially confound our ability to interpret and compare results between studies. Furthermore, differences in each study’s implementation of ICA, such as higher or lower model order impacts key characteristics of the derived ICNs, such as their granularity (Mirzaeian et al., 2025). As model order increases, functional units tend to branch out into smaller units (Abou-Elseoud et al., 2010). It is possible that FC between some ICNs derived from lower model orders which are spatially large, spanning multiple networks, may tend to zero out the effects of smaller regions within them as they average across a larger area and move toward a global baseline. For example, in Rong et al. (2023), OT and OC networks were combined into a single visual network, potentially weakening the observed effects and contributing to the null results observed in the visual network, which was an area with some of the most consistently reported aberrations across studies. Similarly, in Kwak et al. (2021), the cerebellum, temporal lobe, and parietal lobe were all represented by a single component, potentially confounding some of the cerebellum-related results with those of cortical regions. These inconsistencies in functional entities make it difficult to interpret results within a common framework and highlight one of the limitations of the current review. Indeed, Cai et al. (2024) displayed the greatest inconsistency with other studies in the analysis and was also assessed as having the lowest relevance to the current study due to more ambiguous reporting of ROIs as well as significant variations in its methodological approach. Similarly, Forlim et al. (2020) and Rong et al. (2023) were rated as having only moderate relevance to the current study because they made relatively limited comparisons which neglected cerebellar (Forlim et al., 2020) and subcortical (Rong et al., 2023) regions in their analyses. It is plausible that the low similarity of findings in these three studies is largely reflective of a lack of information due to their less comprehensive analyses. This observation may underscore the importance of future studies focusing efforts on more detailed reporting of their ROIs as well as employing more comprehensive data-driven whole-brain approaches.

### Insights, limitations, and recommendations

The current review also yielded many valuable insights into different subdomains within the brain. For example, the paralimbic subdomain (PL) was the subdomain with the most null results across studies, primarily because relatively few studies (Cai et al., 2024; Gong et al., 2019; Kwak et al., 2021; Zarghami et al., 2023) reported ROIs which overlapped with these regions. Notably, the spatial area covered by the PL is relatively small and may require the increased granularity offered by higher model orders, possibly explaining why it is less commonly delineated in existing literature. In contrast to the PL, there were some networks, such as those within the triple network domain (TN) and frontal subdomain (FR), which were frequently implicated across studies and yet also seemed to yield a lower net study count (see Figure 4) due to inconsistent findings. One possibility is that networks incorporating anterior association cortex, such as these, are more frequently involved in higher cognitive processes and are more likely to vary across individuals and studies than more primal networks like motor and sensory cortex (e.g., visual, auditory, and somatosensory; Mueller et al., 2013; Sun et al., 2022). Indeed, higher cognitive networks incorporating the prefrontal cortex, such as those within TN and FR, may be more dynamic, with FC varying more over time (Iraji et al., 2019). One limitation of studies examining static FC, is that they may not capture the full range of variability of more dynamic brain networks, and as a result, findings may be less consistent for these networks across studies. Iraji et al. (2024) in the current review may lend support to this notion, as the observed group differences for the CE (or frontoparietal/attention networks) changed across different states. Unfortunately, one limitation of the current review is that it was not sensitive to time-varying changes such as this, but instead was constrained to comparisons of static FC. As dynamic FC approaches are more widely employed to capture these patterns, the field would greatly benefit from a standardized approach to summarizing and comparing dynamic FC across studies.

While all studies included in the review utilized reasonable measures to minimize the effects of head motion as much as possible in data collection, processing, and analysis, it should be noted that there are different methods for accounting for these effects and that none of them may fully eliminate the effects of head motion (Power et al., 2012, 2014). Conversely, methods of head motion artifact removal may also introduce the possibility of eliminating meaningful signal in rs-fMRI (Bright and Murphy, 2015; Kumar et al., 2024). Therefore, differences in methodology for handling motion artifacts may further contribute to varying results reported in the current review and future investigations into these effects are warranted.

Another limitation of the current review was the sample heterogeneity across included studies. In addition, as previously noted, factors of medication and chronicity were largely unaccounted for and underexplored. Furthermore, the datasets which were utilized varied greatly in sample size and some were represented disproportionately across studies. For example, three studies utilized the COBRE dataset (Iraji et al., 2024; Yan et al., 2024; Zarghami et al., 2023). Our approach to summarizing common findings does not fully account for the various methodological and cohort differences across studies, nonetheless, our sensitivity analysis (Supplementary Appendix 2) may suggest that differences in sample sizes and cohort overlap made little difference. Additionally, our approach does not consider the actual strength of observed patterns (e.g., effect size), but instead only reports the frequency with which the patterns were observed across studies. It is crucial for neuroimaging studies to provide all of the essential data needed to promote transparency and reproducibility of research, as well as to enable meta-analytic literature reviews (Nichols et al., 2017). Furthermore, in order to help establish reliable imaging markers, future studies should seek to validate the highlighted patterns of dysconnectivity in large datasets as well as across different stages of the disease such as clinical high-risk, first-episode and early-psychosis, and chronic schizophrenia, while accounting for the effects of medication. In addition, future work is needed to translate these findings into clinically actionable markers. Prior work has sought to solidify the link between functional architecture and underlying chemoarchitecture (Hansen et al., 2022). Following this line of work, it would be useful to move beyond the circuit level and further explore the neurobiological mechanisms of these changes at the molecular and cellular levels through studies designed to identify cell receptors, neurotransmitters, and cytoarchitecture involved in the target brain circuits. The identification of new neurotransmitters involved in altered brain circuits in schizophrenia could inform the development of new treatments, for example, non-dopaminergic antipsychotics (Kaul et al., 2024).

## Conclusion

Andreasen et al. (1998) and many others (Anticevic et al., 2015; Hwang et al., 2022; Menon et al., 2023; Woodward and Heckers, 2016; Zhou et al., 2015) have focused on the prefrontal cortex as a key node in the cognitive dysmetria framework, viewing schizophrenia as a disease of higher cognitive functions. While there is much evidence supporting dysconnectivity in this node and the implicated etiology is feasible, findings are largely inconsistent across studies. This is likely because the complex relationship between this node and others requires more sophisticated analytical approaches, for example dynamic FC approaches which are sensitive to time-varying changes. Therefore, although the prefrontal cortex remains integral to understanding the neurobiological substrate of schizophrenia and psychosis, concentrating on this node may not be effective for establishing stable imaging markers, at least while employing analytical approaches which investigate static FC in rs-fMRI. Instead, adapting new analytical strategies, or focusing on nodes in the cerebellum, thalamus, and primary motor and sensory (e.g., SM, IT, and OC) or possibly more posterior association cortex (e.g., TP and OT) may prove to be a more effective approach. Further investigation is needed to explore how these patterns of dysconnectivity vary in relation to medication and chronicity as well as across individuals with unique clinical profiles within schizophrenia and psychosis spectrum disorders.

## Data Availability

The original contributions presented in the study are included in the article/supplementary material, further inquiries can be directed to the corresponding author.

## Footnotes

^1^Referenced articles were utilized when pre-existing templates were employed.

^2^ http://neurosynth.org/

^3^Fewer FC pairs are possible in studies constrained to larger spatial scales, for example, extended thalamic subdomain (ET) – basal ganglia subdomain (BG) FC cannot be calculated if both of those subdomains are represented by a single ROI.

^4^ https://jbi.global/critical-appraisal-tools

^5^ http://trendscenter.org/software/gift
