# Supplementary material for: Aberrant cortical–subcortical-cerebellar connectivity in resting-state fMRI as an imaging marker of schizophrenia and psychosis: a systematic review of data-driven whole-brain functional connectivity analyses
Source: Front Neuroimaging. 2025 Oct 10;4:1650987. doi: 10.3389/fnimg.2025.1650987 (PMC12549315; doi:10.3389/fnimg.2025.1650987)
Supplement: Supplementary file 1 [file Table_1.DOCX]

**Appendix 1**

**Clinical Associations with FC**

Symptom associations with FC were mixed, with Cai et al. (2024) reporting a positive correlation between DM-ET FC and PANSS general, Du et al. (2020) reporting a negative correlation between CB-ET FC and PANSS negative, Gong et al. (2019) reporting a positive correlation between SM-ET FC and PANSS total, PANSS general, and PANSS negative, and a positive correlation between IT-ET and OT-ET FC and PANSS negative, and a negative correlation between ET-ET FC and PANSS negative. Although results did not survive FDR correction, Rong et al. (2023) reported a positive correlation between FR-SM FC and PANSS total, PANSS positive, and PANSS negative and a negative correlation between DM-DM FC and PANSS general. Three studies utilized the scale for the assessment of negative symptoms (SANS; Andreasen, 1983) and the scale for the assessment of positive symptoms (SAPS; Andreasen, 1984), although only two reported associations between symptom severity and FC (Forlim et al., 2020; Jensen et al., 2024). Forlim et al. (2020) reported a negative correlation between DM-DM FC and SANS composite score as well as the individual item for apathy. Although none of the symptom associations survived FDR correction, Jensen, Calhoun, et al. (2024) reported positive correlations between SANS global and FR-BG FC and SM-EH FC, as well as between SAPS global and FR-BG FC. Jensen, Calhoun, et al. (2024) also reported negative correlations between SANS global and IT-OT FC, IT-FR FC, OC-FR FC, and CB-CB FC, as well as between SAPS global and CB-ET FC and CB-EH FC.

Only two studies tested for associations between duration of illness (DOI) and FC, with Gong et al. (2019) reporting a negative correlation between DOI and inter-thalamic FC and Rong et al. (2023) reporting a negative correlation between DOI and IT-SM FC. Four studies tested for associations between antipsychotic use and FC, with Gong et al. (2019) reporting a negative correlation between CPZ and CB-ET FC and a positive correlation between CPZ and OT-ET FC and SM-ET FC, and Rong et al. (2023) reporting a positive correlation between CPZ and TP-SM FC. Forlim et al. (2020) and Iraji et al. (2024) tested for correlations between CPZ and FC but reported no significant relationships.

Only two studies examined associations between cognitive assessments and FC. Cai et al. (2024) reported a positive correlation between Stockings of Cambridge and FR-ET FC, although this association did not survive FDR correction. Zarghami et al., (2023) reported significant impairments in schizophrenia across all seven MATRICS domains with top CCA results of SM-SM EF and PL-TP EF with top traits of social cognition, reasoning/problem-solving, and working memory.

**Appendix 2**

**Sample Heterogeneity Sensitivity Analysis**

**Table S1** | Datasets and sample sizes for the eight studies included in the sensitivity analysis subset. A) FBIRN: Function Biomedical Informatics Research Network data repository (Keator et al., 2016). B) MPRC: Maryland Psychiatric Research Center (Adhikari et al., 2019). C) COBRE: Center for Biomedical Research Excellence (Aine et al., 2017). D) Demographics are reported separately for the two datasets in Du et al. (2020) because the analyses and results are reported for each dataset separately.

With consideration to possible concerns about the sample heterogeneity across studies and the possibility of an overrepresentation of the COBRE, FBIRN, and MPRC datasets, we performed a sensitivity analysis in which we removed the FC results of Iraji et al. (2024) and Yan et al. (2024) from the study count (see **Figure 3** in the main text). The omission of these two studies resulted in a total of 8 studies, each with non-overlapping datasets and more uniform sample sizes across the included studies, with sample sizes ranging from 76-388 (see **Table S1**).

| Study | Dataset(s) | Total N |
| --- | --- | --- |
| Cai et al. (2024) | West China Hospital Sichuan University | 160 |
| Du et al. (2020) | FBIRN^a^; MPRC^b^ | 281; 388^d^ |
| Forlim et al. (2020) | St. Hedwig Hospital (Erlangen, Germany) | 76 |
| Gong et al. (2019) | University of Electronic Science and Technology of China | 96 |
| Jensen et al. (2024) | University of Pittsburgh; Johns Hopkins | 247 |
| Kwak et al. (2021) | Seoul National University | 80 |
| Rong et al. (2023) | Wuhan University | 365 |
| Zarghami et al. (2023) | COBRE^c^ | 140 |

Although there were two fewer studies lending support to the most prominent patterns of FC across studies, the overall patterns remained mostly unchanged (see **Figure S1**), with a Pearson correlation of *r* = .91 (*p* < .05) between the net study counts calculated with the eight studies and with all 10 studies.


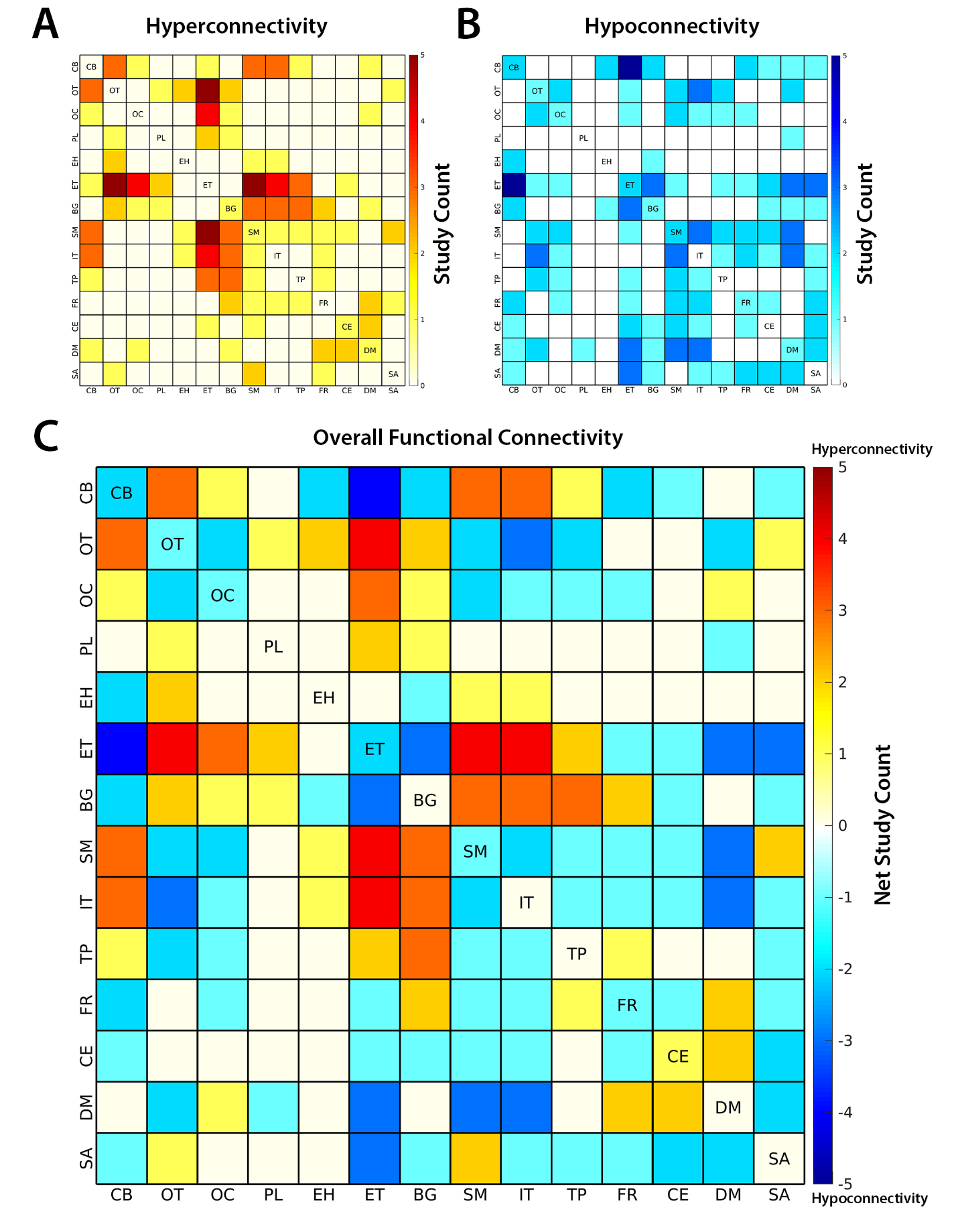


**Figure S1** | The number of studies reporting a) hyperconnectivity and b) hypoconnectivity between functional subdomains of the brain are shown above. Hyperconnectivity represents an increase in the positive directionality of functional connectivity (FC) in schizophrenia (SZ) relative to controls and hypoconnectivity represents a relative decrease (or increase in negative directionality) in SZ. The overall patterns of FC are represented by net study count, where studies reporting hyperconnectivity are assigned +1 and studies reporting hypoconnectivity are assigned -1. The 14 subdomains are based on the NeuroMark 2.2 multi-scale template: cerebellar (CB), visual-occipitotemporal (OT), visual-occipital (OC), paralimbic (PL), subcortical-extended hippocampal (EH), subcortical-extended thalamic (ET), subcortical-basal ganglia (BG), sensorimotor (SM), higher cognition-insular temporal (IT), higher cognition-temporoparietal (TP), higher cognition-frontal (FR), triple network-central executive (CE), triple network-default mode (DM), and triple network-salience (SA).

**Figure 3** | The number of studies reporting a) hyperconnectivity and b) hypoconnectivity between functional subdomains of the brain are shown above. Hyperconnectivity represents an increase in the positive directionality of functional connectivity (FC) in schizophrenia (SZ) relative to controls and hypoconnectivity represents a relative decrease (or increase in negative directionality) in SZ. The overall patterns of FC are represented by net study count, where studies reporting hyperconnectivity are assigned +1 and studies reporting hypoconnectivity are assigned -1. The 14 subdomains are based on the NeuroMark 2.2 multi-scale template: cerebellar (CB), visual-occipitotemporal (OT), visual-occipital (OC), paralimbic (PL), subcortical-extended hippocampal (EH), subcortical-extended thalamic (ET), subcortical-basal ganglia (BG), sensorimotor (SM), higher cognition-insular temporal (IT), higher cognition-temporoparietal (TP), higher cognition-frontal (FR), triple network-central executive (CE), triple network-default mode (DM), and triple network-salience (SA).

**References**

Adhikari, B. M., Hong, L. E., Sampath, H., Chiappelli, J., Jahanshad, N., Thompson, P. M., Rowland, L. M., Calhoun, V. D., Du, X., Chen, S., & Kochunov, P. (2019). Functional network connectivity impairments and core cognitive deficits in schizophrenia. *Human Brain Mapping*, *40*(16), 4593–4605. https://doi.org/10.1002/hbm.24723

Aine, C. J., Bockholt, H. J., Bustillo, J. R., Cañive, J. M., Caprihan, A., Gasparovic, C., Hanlon, F. M., Houck, J. M., Jung, R. E., Lauriello, J., Liu, J., Mayer, A. R., Perrone-Bizzozero, N. I., Posse, S., Stephen, J. M., Turner, J. A., Clark, V. P., & Calhoun, V. D. (2017). Multimodal Neuroimaging in Schizophrenia: Description and Dissemination. *Neuroinformatics*, *15*(4), 343–364. https://doi.org/10.1007/s12021-017-9338-9

Andreasen, N. C. (1983). *The Scale for the Assessment of Negative Symptoms (SANS)*. Iowa City, Iowa: The University of Iowa.

Andreasen, N. C. (1984). *The Scale for the Assessment of Positive Symptoms (SAPS)*. Iowa City, Iowa: The University of Iowa.

Cai, J., Xie, M., Liang, S., Gong, J., Deng, W., Guo, W., Ma, X., Sham, P. C., Wang, Q., & Li, T. (2024). Dysfunction of thalamocortical circuits in early-onset schizophrenia. *Cerebral Cortex*, *34*(8), bhae313. https://doi.org/10.1093/cercor/bhae313

Du, Y., Fu, Z., Sui, J., Gao, S., Xing, Y., Lin, D., Salman, M., Abrol, A., Rahaman, M. A., Chen, J., Hong, L. E., Kochunov, P., Osuch, E. A., & Calhoun, V. D. (2020). NeuroMark: An automated and adaptive ICA based pipeline to identify reproducible fMRI markers of brain disorders. *NeuroImage: Clinical*, *28*, 102375. https://doi.org/10.1016/j.nicl.2020.102375

Forlim, C. G., Klock, L., Bächle, J., Stoll, L., Giemsa, P., Fuchs, M., Schoofs, N., Montag, C., Gallinat, J., & Kühn, S. (2020). Reduced Resting-State Connectivity in the Precuneus is correlated with Apathy in Patients with Schizophrenia. *Scientific Reports*, *10*(1), 2616. https://doi.org/10.1038/s41598-020-59393-6

Gong, J., Luo, C., Li, X., Jiang, S., Khundrakpam, B. S., Duan, M., Chen, X., & Yao, D. (2019). Evaluation of functional connectivity in subdivisions of the thalamus in schizophrenia. *The British Journal of Psychiatry*, *214*(5), 288–296. https://doi.org/10.1192/bjp.2018.299

Iraji, A., Chen, J., Lewis, N., Faghiri, A., Fu, Z., Agcaoglu, O., Kochunov, P., Adhikari, B. M., Mathalon, D. H., Pearlson, G. D., Macciardi, F., Preda, A., Van Erp, T. G. M., Bustillo, J. R., Díaz-Caneja, C. M., Andrés-Camazón, P., Dhamala, M., Adali, T., & Calhoun, V. D. (2024). Spatial Dynamic Subspaces Encode Sex-Specific Schizophrenia Disruptions in Transient Network Overlap and Their Links to Genetic Risk. *Biological Psychiatry*, *96*(3), 188–197. https://doi.org/10.1016/j.biopsych.2023.12.002

Jensen, K. M., Calhoun, V. D., Fu, Z., Yang, K., Faria, A. V., Ishizuka, K., Sawa, A., Andrés-Camazón, P., Coffman, B. A., Seebold, D., Turner, J. A., Salisbury, D. F., & Iraji, A. (2024). A whole-brain neuromark resting-state fMRI analysis of first-episode and early psychosis: Evidence of aberrant cortical-subcortical-cerebellar functional circuitry. *NeuroImage: Clinical*, *41*, 103584. https://doi.org/10.1016/j.nicl.2024.103584

Keator, D. B., Van Erp, T. G. M., Turner, J. A., Glover, G. H., Mueller, B. A., Liu, T. T., Voyvodic, J. T., Rasmussen, J., Calhoun, V. D., Lee, H. J., Toga, A. W., McEwen, S., Ford, J. M., Mathalon, D. H., Diaz, M., O’Leary, D. S., Jeremy Bockholt, H., Gadde, S., Preda, A., … Potkin, S. G. (2016). The Function Biomedical Informatics Research Network Data Repository. *NeuroImage*, *124*, 1074–1079. https://doi.org/10.1016/j.neuroimage.2015.09.003

Kwak, Y. B., Cho, K. I. K., Hwang, W. J., Kim, A., Ha, M., Park, H., Lee, J., Lee, T. Y., Kim, M., & Kwon, J. S. (2021). Mapping thalamocortical functional connectivity with large-scale brain networks in patients with first-episode psychosis. *Scientific Reports*, *11*(1), 19815. https://doi.org/10.1038/s41598-021-99170-7

Rong, B., Huang, H., Gao, G., Sun, L., Zhou, Y., Xiao, L., Wang, H., & Wang, G. (2023). Widespread Intra- and Inter-Network Dysconnectivity among Large-Scale Resting State Networks in Schizophrenia. *Journal of Clinical Medicine*, *12*(9), 3176. https://doi.org/10.3390/jcm12093176

Yan, W., Pearlson, G. D., Fu, Z., Li, X., Iraji, A., Chen, J., Sui, J., Volkow, N. D., & Calhoun, V. D. (2024). A Brainwide Risk Score for Psychiatric Disorder Evaluated in a Large Adolescent Population Reveals Increased Divergence Among Higher-Risk Groups Relative to Control Participants. *Biological Psychiatry*, *95*(7), 699–708. https://doi.org/10.1016/j.biopsych.2023.09.017

Zarghami, T. S., Zeidman, P., Razi, A., Bahrami, F., & Hossein‐Zadeh, G. (2023). Dysconnection and cognition in schizophrenia: A spectral dynamic causal modeling study. *Human Brain Mapping*, *44*(7), 2873–2896. https://doi.org/10.1002/hbm.26251
